# Establishment of a High-Efficiency In Vitro Regeneration and *Agrobacterium*-Mediated Genetic Transformation System for *Ajania achilleoides* (Turcz.) Poljakov

**DOI:** 10.3390/biotech15030059

**Published:** 2026-07-28

**Authors:** Xiaoyue Zheng, Hao Li, Shu Wang, Huiyan Cheng, Huien Zhao

**Affiliations:** National Engineering Research Center for Floriculture, School of Landscape Architecture, Beijing Forestry University, Beijing 100083, China; c_xiaoyuezheng@163.com (X.Z.); lihao818816@163.com (H.L.); wangshu809@bjfu.edu.cn (S.W.); chenghuiyan1913@gmail.com (H.C.)

**Keywords:** *Ajania achilleoides*, in vitro morphogenesis, *Agrobacterium*-mediated transformation, hygromycin selection, adventitious shoot induction

## Abstract

*Ajania achilleoides*, a wild species with strong environmental adaptability, is a valuable germplasm resource for genetic improvement and functional gene studies. However, efficient in vitro regeneration and genetic transformation systems for this species remain undeveloped, limiting functional gene characterization and genetic improvement of this valuable germplasm resource. In this study, leaf explants were used to establish an efficient regeneration system by evaluating different combinations of plant growth regulators and to develop an *Agrobacterium* tumefaciens-mediated genetic transformation system for *A. achilleoides*. MS medium supplemented with 2.0 mg/L 6-benzyladenine (6-BA) and 1.0 mg/L α-naphthaleneacetic acid (NAA) achieved the highest adventitious shoot regeneration rate (99%), while 1/2 MS medium containing 0.5 mg/L indole-3-butyric acid (IBA) was optimal for rooting, resulting in a 100% rooting rate. Critical hygromycin concentrations for shoot regeneration and rooting selection were determined to be 25 mg/L and 10 mg/L, respectively. For transformation, the best results were obtained with a 3-day preculture, bacterial suspension at OD_600_ = 0.2, 30 min infection, and 2-day co-cultivation. PCR and sequencing confirmed the successful integration of the target genes into the genome, yielding three positive transgenic lines with a molecular confirmation rate of 30%. This study establishes a stable and efficient regeneration and transformation system for *A. achilleoides*, providing a platform for functional gene analysis and molecular breeding of wild chrysanthemum species.

## 1. Introduction

*Ajania achilleoides* is a perennial subshrub belonging to the genus *Ajania* of the tribe Anthemideae in the family Asteraceae. It is mainly distributed in the desert-steppe ecosystems of northern China and Mongolia [1]. Owing to its long-term adaptation to arid environments, this species has evolved strong drought tolerance and resistance to nutrient-poor conditions, making it a valuable resource for ecological restoration, forage utilization, medicinal exploitation, and ornamental horticulture [2,3,4]. In addition, *A. achilleoides* is phylogenetically closely related to cultivated chrysanthemum and is therefore regarded as an important wild germplasm resource for improving stress resistance in chrysanthemum breeding programs [5,6]. However, the efficient utilization of its desirable genetic resources remains limited by the low efficiency of conventional hybrid breeding and the complex genetic background of chrysanthemum-related species. Therefore, the establishment of an efficient in vitro regeneration and genetic transformation system for *A. achilleoides* is of great significance for functional characterization of stress-responsive genes and molecular breeding applications.

Efficient genetic transformation in plants is highly dependent on a stable regeneration system. The tribe Anthemideae contains several economically important genera, including *Chrysanthemum*, *Ajania*, and *Artemisia*, which contain species of value for ornamental improvement, secondary metabolite research, stress adaptation, and plant resource utilization. Therefore, in vitro regeneration studies in related taxa can provide useful references for establishing a regeneration system for *A. achilleoides*. In recent years, reliable in vitro regeneration protocols have been developed for several species within the tribe Anthemideae. In cultivated chrysanthemum, regeneration systems using *Chrysanthemum* ‘11-C-2’ and ‘Shinma’ as materials have been optimized for adventitious shoot regeneration, rooting, and related culture conditions [7,8]. In other related taxa, studies on *Chrysanthemum vestitum* and *Artemisia annua* have focused on regeneration system establishment using different plant materials and the effects of in vitro regeneration on secondary metabolism, respectively [9,10]. In *Ajania* species more closely related to *A. achilleoides*, callus induction, plant regeneration, and rapid propagation through tissue culture have been reported in *Ajania purpurea*, *Aj. scharnhorstii*, and *Aj. alabasica* [11,12,13]. Studies on ornamental plants and related Asteraceae taxa have demonstrated that the type and concentration of plant growth regulators, their combinations, and the composition of culture media significantly affect callus induction and adventitious shoot regeneration efficiency [14,15,16,17,18]. Nevertheless, substantial interspecific variation in regeneration capacity exists, and regeneration protocols established for one species are often not directly transferable to other species. To our knowledge, a systematic in vitro regeneration protocol has not yet been reported for A. achilleoides. Consequently, developing an efficient regeneration system for *A. achilleoides* represents a prerequisite for subsequent genetic transformation studies.

*Agrobacterium*-mediated transformation has been widely applied in functional genomics and genetic improvement because of its simplicity, high stability, and reproducibility [19,20]. Previous studies have shown that transformation efficiency is significantly influenced by factors such as bacterial suspension density, pre-culture duration, infection duration, co-cultivation period, and selection strategy [21]. In this process, an effective in vitro regeneration system is essential because transformed cells must retain organogenic competence and regenerate into complete plants after infection and selection. To date, *Agrobacterium*-mediated transformation systems have been established in several species of the tribe Anthemideae and related Asteraceae plants. For example, Akram et al. established an *Agrobacterium*-mediated transformation method based on direct regeneration in chamomile (*Matricaria chamomilla*) [22], and Shinoyama et al. developed a genetic transformation system for pyrethrum (*Tanacetum cinerariifolium*) [23]. In addition, plant regeneration, *Agrobacterium*-mediated transformation, and T-DNA insertion site identification have also been reported in ornamental chrysanthemum (*Chrysanthemum morifolium*) and the wild chrysanthemum model species *Chrysanthemum seticuspe* [24,25]. However, these studies have focused primarily on cultivated chrysanthemum, whereas stable and efficient transformation protocols for wild *Ajania* species remain scarce, particularly for *A. achilleoides*. This limitation has hindered the molecular validation and functional characterization of candidate stress-resistance genes in this species, thereby restricting the effective utilization of its wild germplasm resources.

Therefore, the present study systematically investigated the key factors affecting both in vitro regeneration and *Agrobacterium*-mediated transformation of *A. achilleoides*, including plant growth regulator combinations, *Agrobacterium* infection parameters, and selection conditions. The objective was to establish a stable, efficient, and reproducible regeneration and transformation system, thereby providing a technical platform for functional analysis of stress-related genes and molecular breeding of *A. achilleoides*.

## 2. Materials and Methods

### 2.1. Plant Materials and Culture Conditions

The plant material used in this study was *A. achilleoides* collected from Inner Mongolia by Prof. Huien Zhao and preserved at the Plant Science Center of Beijing Forestry University. Bud-bearing stem segments were initially used as explants for the establishment of a sterile system. After surface sterilization and culture establishment, the resulting in vitro-grown plantlets were maintained under controlled conditions at 20–22 °C, with a fluorescent light source at an intensity of 54 μmol·m^−2^·s^−1^, and a photoperiod of 16 h light/8 h dark. Leaves and bud-bearing stem segments from these in vitro-grown plantlets were subsequently used as explants for regeneration experiments.

### 2.2. Establishment of Sterile System

Bud-bearing stem segments of *A. achilleoides* were collected and used for the establishment of a sterile system. The segments were washed with detergent solution using a soft brush, placed into wide-mouth bottles, covered with gauze, and rinsed under running tap water for 2 h. Sterilization was conducted on a laminar flow bench as follows: 75% ethanol for 30 s, rinsed twice with sterile water for 2 min each, followed by 2% (*w*/*v*) sodium hypochlorite solution for 3 min, and rinsed three times with sterile water for 3 min each. After surface sterilization, the cut surfaces were trimmed on sterile filter paper, and the stem segments were inoculated onto half-strength Murashige and Skoog (MS) medium (PhytoTech Labs, Lenexa, KS, USA) containing 50% MS salts, full-strength MS vitamins, 30 g L^−1^ sucrose, and 7 g L^−1^ agar without plant growth regulators (PGRs).

### 2.3. Callus Induction and Adventitious Bud Differentiation

MS medium supplemented with different concentrations of 6-benzyladenine (6-BA; Solarbio, Beijing, China) and α-naphthaleneacetic acid (NAA; Solarbio, Beijing, China) was used for callus induction and adventitious bud differentiation. The tested concentrations of 6-BA were 1, 2, 3 mg/L, while NAA concentrations were 0.5, 0.8, 1.0 mg/L. Fully expanded, healthy, sterile seedling leaves were cut into 0.5 cm × 0.5 cm leaf discs and placed adaxial side up on the medium. Each culture medium formulation contained 20 explants, with three replicates per treatment. Explants were cultured at 20–22 °C under a fluorescent light intensity of 54 μmol·m^−2^·s^−1^ with a photoperiod of 16 h light/8 h dark. Callus formation and adventitious bud differentiation were observed and photographed every 15 days. Callus induction was evaluated based on the appearance of callus tissues from explants, while organogenesis was determined by the emergence of visible adventitious buds from callus tissues. The callus induction rate and bud induction rate were calculated and recorded.

### 2.4. Rooting Culture

Adventitious buds over 1–2 cm in length were transferred to rooting induction media (Table 1). Each medium formulation was tested in three replicates, with 15 buds per replicate and 3 buds per bottle. Culture conditions were the same as described above. After 30 days, the rooting rate, average root number, and average root length were recorded.

### 2.5. Acclimatization and Transplantation

Regenerated plantlets after 30 days of rooting culture with established root systems were acclimated by opening the vessel lids for 3 days and then transplanted into a 1:1 (*v*/*v*) peat mixed substrate. Plants were maintained in a growth chamber, covered with a humidity dome for the first 7 days with occasional misting to prevent desiccation, then uncovered. Seedling survival rate was recorded after 15 days. The experiment was repeated three times. Seedlings were grown in a greenhouse under near-natural conditions.

### 2.6. Hygromycin Sensitivity Test

To optimize the genetic transformation of *A. achilleoides*, leaf and adventitious bud sensitivity to hygromycin B (Solarbio, Beijing, China) was assessed to guide the selection of positive transformants. The previously optimized callus induction and differentiation medium was used as the basal medium. Leaves were inoculated on medium containing 0, 5, 10, 15, 20 or 25 mg/L hygromycin. Culture conditions were as described above. Leaf differentiation responses, including differentiation rate and growth status, were evaluated, and one-way ANOVA followed by LSD multiple comparison tests was performed to determine significant differences among hygromycin treatments. Based on these results, the appropriate hygromycin concentration for leaf selection was determined.

Adventitious buds (1–2 cm) were excised with a scalpel and inoculated onto rooting medium containing 0, 5, 10, 15, 20 mg/L hygromycin. Root induction was conducted for 30 days, and the rooting number, root length, and rooting rate were recorded. Each treatment included 5 plates with 10 buds per plate. The appropriate hygromycin concentration for adventitious bud selection was determined based on rooting responses and statistical analysis.

### 2.7. Preparation of Agrobacterium Suspension

**Figure 1:**
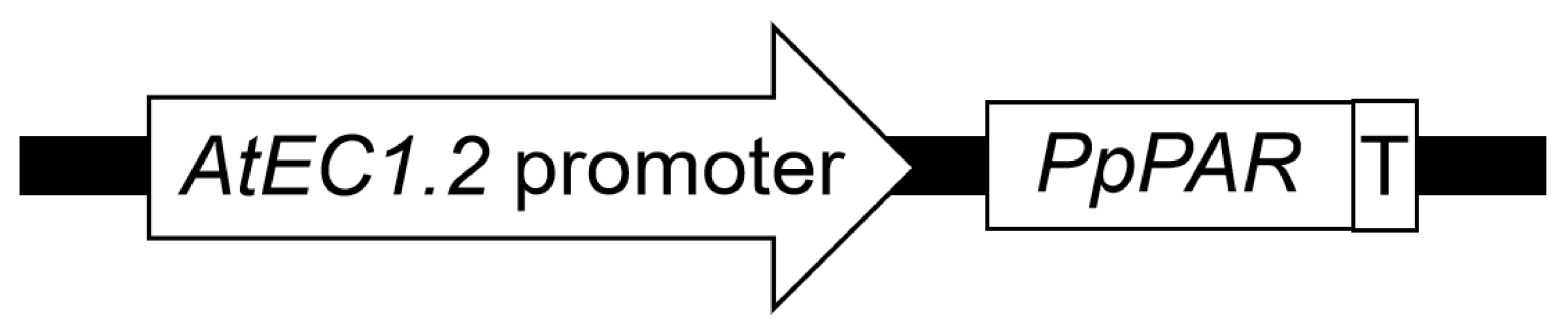
Structure of plant expression vector pC1300 (The vector was kindly provided by Prof. Kejian Wang and his research group at the China National Rice Research Institute (CNRRI), Chinese Academy of Agricultural Sciences, Beijing, China).

EHA105 *Agrobacterium tumefaciens* containing the plant expression vector pC1300 (Figure 1) was removed from −80 °C storage and thawed on ice. A small amount of bacterial suspension was streaked onto 100 mL LB agar (Solarbio, Beijing, China) containing 50 mg/L kanamycin (Kan; Solarbio, Beijing, China) and 50 mg/L rifampicin (Rif; Solarbio, Beijing, China) under sterile conditions. Plates were incubated upside down at 2 °C for 2 days until single colonies appeared. A single colony was inoculated into liquid LB containing Kan and Rif and cultured on a shaker at 2 °C until turbid.

PCR was used to verify the presence of the *Arabidopsis thaliana* egg cell-specific promoter *AtEC1.2* and *Pilosella piloselloides* gene *PAR*. Positive colonies were scaled up at a 1:100 ratio, centrifuged at 3000× *g* for 10 min, and resuspended in MES solution (2-(N-morpholino)ethanesulfonic acid; Solarbio, Beijing, China).

### 2.8. Screening of Genetic Transformation System

Factors affecting genetic transformation were evaluated, including preculture duration, bacterial suspension concentration (OD_600_), infection time, and coculture duration. Each factor had four levels (Table 2), resulting in 16 treatment combinations, each with three replicates of approximately 60 leaf discs per replicate. After 30 days, callus induction rate, differentiation rate, adventitious bud formation coefficient, and browning rate were recorded.

Leaf discs were precultured on the selected optimal medium for 3, 2, 1, or 0 days. Bacterial suspensions were resuspended in MES and adjusted to OD_600_ values of 0.2, 0.4, 0.6, and 0.8. Leaf discs were infected in bacterial suspensions for 5, 10, 20, or 30 min with continuous gentle shaking to ensure full contact.

After infection, excess bacterial suspension was removed with filter paper, and leaf discs were placed on the optimal regeneration medium for coculture in the dark for 1, 2, 3, or 4 days. After coculture, leaf discs were transferred to selection medium containing 25 mg/L hygromycin and 350 mg/L timentin.

### 2.9. Transgene Detection

Primers used for PCR detection of the egg cell-specific promoter (*AtEC1.2*) promoter and PpPAR gene were synthesized by Beijing Qingke Biotechnology Co., Ltd. (Beijing, China). The primer sequences used for amplification are listed in Table 3. ddH2O, wild-type plant DNA, and plasmid DNA were used as blank, negative, and positive controls, respectively.

PCR reactions were performed in a 20 μL volume containing 1 μL DNA template, 1 μL of each primer, 10 μL 2× PCR mix (Vazyme Biotech Co., Ltd., Nanjing, China), and ddH2O to volume. Thermal cycling conditions were: 95 °C for 3 min; 28 cycles of 95 °C for 15 s, 58 °C for 15 s, 72 °C for 15 s; followed by 72 °C for 5 min.

PCR-positive products were purified and submitted to Sanger sequencing by Kangwei Century Biotechnology Co., Ltd. (Taizhou City, China). The sequencing chromatograms were examined, and the obtained sequences were aligned with the expected *AtEC1.2* promoter and *PpPAR* gene sequences. Sequence alignment analysis confirmed that the amplified fragments from resistant plantlets corresponded to the target transgene sequences.

### 2.10. Data Analysis

Data were processed using Microsoft Excel 2019 (Microsoft Corporation, Redmond, WA, USA). Orthogonal experimental designs were applied for the establishment of the regeneration system and *Agrobacterium*-mediated transformation conditions, including the evaluation of multiple factors and levels. For orthogonal experiments, range analysis was performed to evaluate the relative effects of different factors and levels on regeneration and resistant shoot regeneration. The range value (R) was calculated to determine the relative contribution of each factor. For single-factor experiments, including hygromycin sensitivity testing and rooting analysis, a completely randomized design was used. Statistical analyses were performed using IBM SPSS Statistics 26 (IBM Corp., Armonk, NY, USA). Analysis of variance (ANOVA) was conducted to evaluate significant differences among treatments, followed by LSD multiple comparison tests when appropriate (*p* < 0.05). The following formulas were used to calculate relevant indices:

Callus induction rate (%) = (number of explants forming callus/total explants) × 100

Adventitious bud differentiation rate (%) = (number of explants forming buds/total explants) × 100

Adventitious bud formation coefficient = total number of buds/number of explants forming buds

Rooting rate (%) = (number of rooted buds/total inoculated buds) × 100

Survival rate after transplantation (%) = (number of surviving plants/total transplanted plants) × 100

Browning rate (%) = (number of browned explants/total explants) × 100

Molecular confirmation rate (%) = (number of positive plants/number of resistant plants) × 100

## 3. Results

### 3.1. Establishment of an In Vitro Morphogenesis System of A. achilleoides

#### 3.1.1. Effects of Plant Growth Regulators on Callus Induction

**Figure 2:**
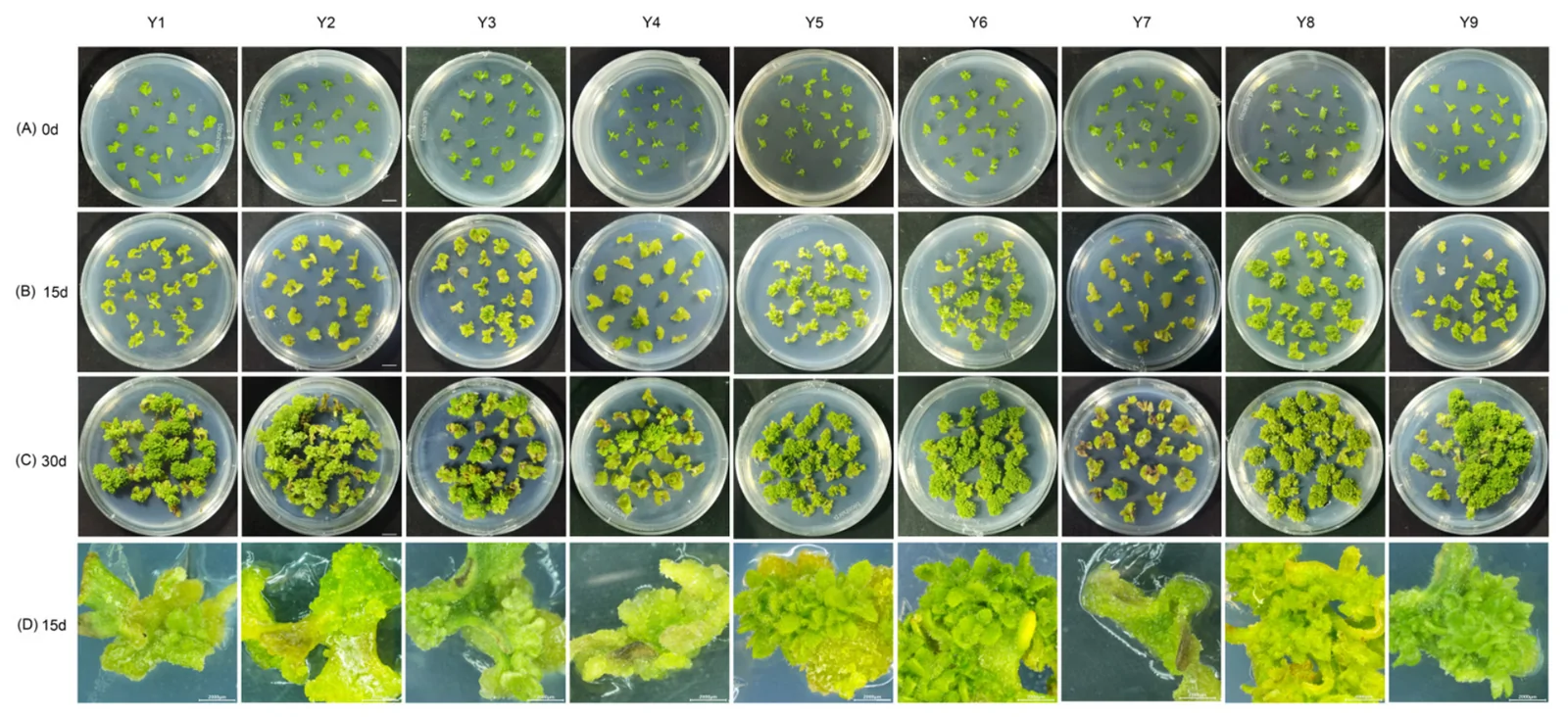
Effects of different medium formulas and different culture time on callus induction and adventitious bud differentiation of *A. achilleoides* leaves. (**A**–**C**) Growth of *A. achilleoides* leaf explants on Y1–Y9 media at 0, 15, and 30 days, respectively. Bars = 1 cm; (**D**) Microscopic observation of *A. achilleoides* leaf explants at 15 days. Bars = 2000 μm.

Different concentration combinations of 6-BA and NAA significantly affected callus induction from explants (Table 4; Figure 2). Range analysis revealed that NAA had a stronger effect than 6-BA, indicating it is the primary factor controlling callus formation (Table 5). Increasing NAA concentration from 0.5 mg/L to 0.8 mg/L and 1.0 mg/L increased the callus induction rate from 75.66% to 98.89% and 100.00%, respectively. No significant difference was observed between the latter two treatments, but both were markedly higher than the 0.5 mg/L treatment. Variation among different 6-BA concentrations was relatively small, with mean induction rates maintained between 88.89% and 92.86%.

Analysis of 6-BA/NAA ratios indicated that lower ratios (1.0–2.0) consistently resulted in higher induction rates. Specifically, treatments of 6-BA 1.0 mg/L + NAA 1.0 mg/L (1:1), 6-BA 2.0 mg/L + NAA 1.0 mg/L (2:1), and 6-BA 3.0 mg/L + NAA 1.0 mg/L (3:1) achieved full (100%) callus induction. After 15 days of culture, explants treated with higher NAA concentrations (treatments 3, 6, and 9 in Table 4) expanded more rapidly, initiated callus formation earlier, and exhibited vigorous growth, whereas low NAA treatments showed slower callus development. These results suggest that under the present experimental conditions, increasing NAA concentration favors higher callus induction rates.

#### 3.1.2. Effects of Plant Growth Regulators on Adventitious Bud Differentiation

The combination of 6-BA and NAA significantly influenced indirect organogenesis and adventitious shoot regeneration from calli (Table 4). Range analysis confirmed that NAA is the main determinant for bud differentiation (Table 5). Differentiation rates increased significantly with higher NAA concentrations, while among 6-BA treatments, 1.0 mg/L and 2.0 mg/L yielded higher mean differentiation than 3.0 mg/L.

Significant differences in differentiation were observed among different 6-BA/NAA combinations. Treatment 6-BA 2.0 mg/L + NAA 1.0 mg/L (2:1) achieved the highest differentiation rate (100%), whereas 6-BA 3.0 mg/L + NAA 0.5 mg/L (6:1) exhibited the lowest rate. Overall, a 6-BA/NAA ratio of 1:1–2:1 was optimal for high adventitious bud differentiation.

After 30 days of culture, explants under Y5 and Y6 treatments showed substantial growth, with Y6 producing dense, uniformly developed, and bright green buds. High 6-BA treatments (treatment 7–9) led to partial morphological abnormalities and restricted growth (Figure 2). Considering both differentiation efficiency and bud quality, Y6 was identified as the optimal treatment for adventitious bud induction.

#### 3.1.3. Effects of Plant Growth Regulators on Rooting

**Figure 3:**
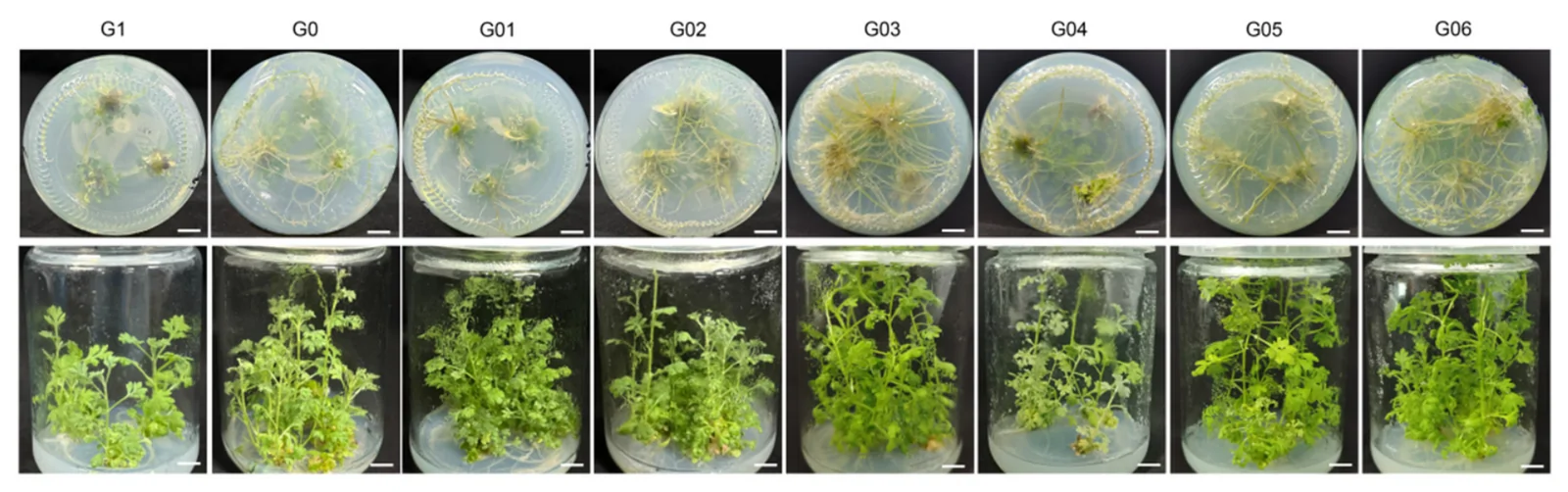
Growth status of roots and plants of *A. achilleoides* in different media for 30 days, bars = 1 cm.

Rooting of adventitious buds was strongly influenced by both the culture medium and plant growth regulator treatment (Table 6; Figure 3). In the absence of exogenous auxins, rooting on half-strength MS medium (1/2 MS) reached 100.00%, higher than the 83.33% on full-strength MS medium. Plants cultured on 1/2 MS exhibited better shoot growth and well-developed root systems, and thus ½ MS was used as the basal medium for subsequent rooting experiments.

Addition of NAA or IBA to 1/2 MS maintained high rooting rates (94.44–100.00%), indicating both auxins were effective in promoting adventitious bud rooting. Differences in root number and root length were more pronounced than in rooting rate. Under NAA, increasing the concentration from 0.1 mg/L to 0.5 mg/L increased the mean root number from 9.44 to 13.33 but decreased the average root length from 10.40 cm to 5.50 cm. In contrast, IBA treatments produced more numerous roots (18.33–19.67), significantly exceeding NAA treatments. Among them, G04 (0.1 mg/L IBA) produced the highest root number (19.67), and G06 (0.5 mg/L IBA) yielded the longest roots (9.07 cm) with a 100% rooting rate.

Morphological observations were consistent with statistical data. NAA-induced roots were fewer, and high NAA (G03) resulted in short, thick roots with limited lateral branches, whereas IBA treatments produced numerous, well-branched, and elongated roots, forming a dense fibrous network. Additionally, G05 and G06 treatments promoted vigorous shoot growth, with overall plant performance superior to other treatments.

Considering the rooting rate, root number, root length, and plant growth, IBA treatments outperformed NAA. Specifically, 1/2 MS + 0.5 mg/L IBA (G06) achieved the highest rooting efficiency, the longest roots, and a well-developed lateral root system, and is recommended as the optimal medium for in vitro micropropagation.

#### 3.1.4. Acclimatization and Transplantation of Regenerated Plantlets

Regenerated plantlets obtained from the optimal regeneration medium (MS +2.0 mg/L 6-BA + 1.0 mg/L NAA) and subsequently rooted on the optimal rooting medium (1/2 MS + 0.5 mg/L IBA) were used for acclimatization and transplantation. These regenerated plantlets were derived from non-transformed wild-type explants and were used to evaluate the completeness and stability of the in vitro morphogenesis system. Fifteen days after transplantation, a survival rate of 72% was achieved. The regenerated plantlets of *A. achilleoides* exhibited vigorous growth and good adaptability to ex vitro conditions. Flower buds were observed 60 days after transplantation, indicating successful establishment and normal development of regenerated wild-type plants.

**Figure 4:**
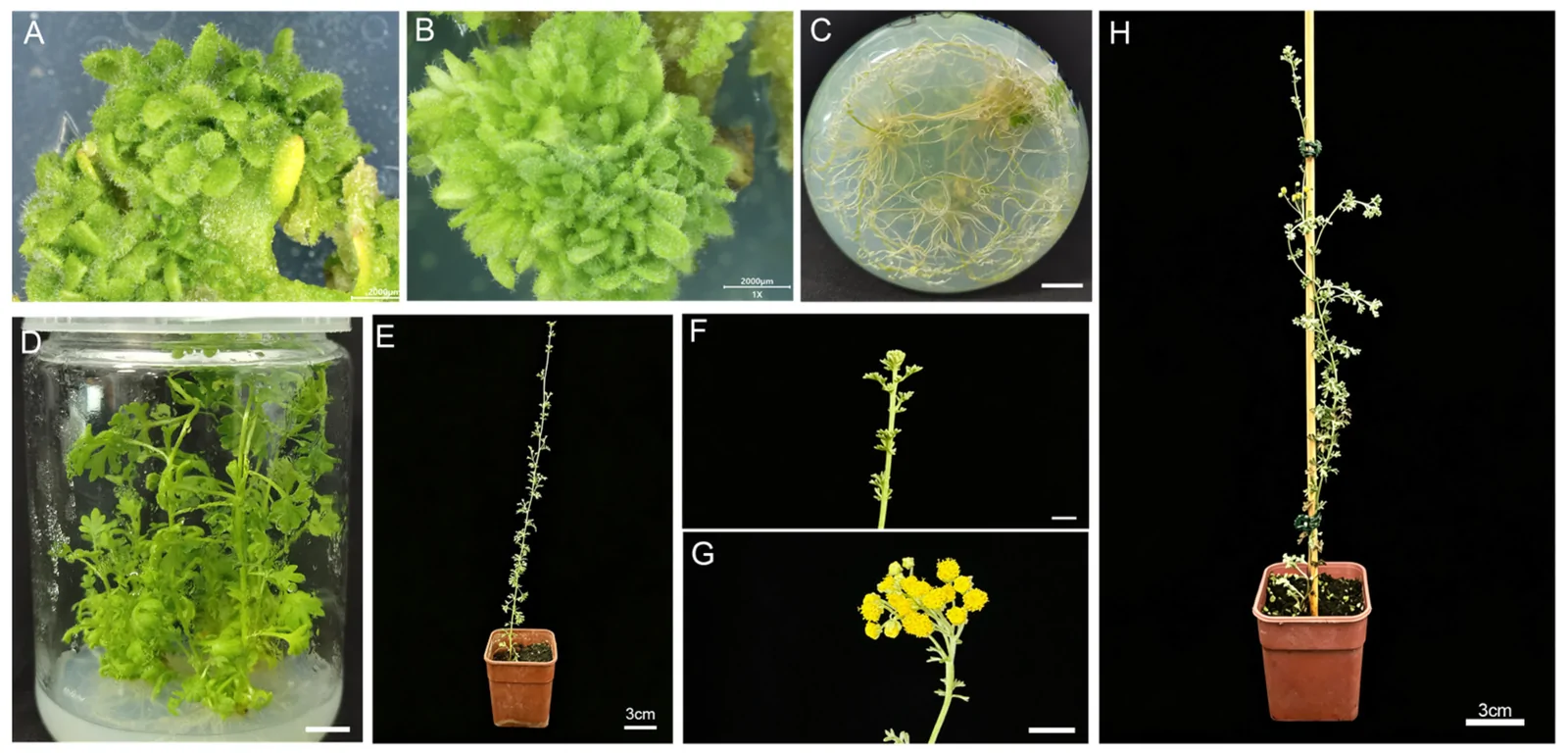
Leaf regeneration system of *A. achilleoides.* (**A**,**B**) Callus induction and adventitious bud differentiation in Y6 medium at 15 and 30 days, bars = 2000 μm; (**C**,**D**) Root and plant status in rooting culture of G06 for 30 days; (**E**,**F**) Plant status and budding after 60 days of transplanting; (**G**,**H**) Flowering plants at 85 days after transplanting. (**C**), (**D**,**F**,**G**) Bars = 1 cm; (**E**,**H**) Bars = 3 cm.

Through sequential callus induction, adventitious bud differentiation, rooting, acclimatization, and transplantation, complete regenerated plants were obtained from leaf explants of *A. achilleoides*, confirming the effectiveness of the established in vitro morphogenesis system (Figure 4).

### 3.2. Establishment of an Agrobacterium tumefaciens-Mediated Transformation System of A. achilleoides

#### 3.2.1. Hygromycin Sensitivity Evaluation

**Figure 5:**
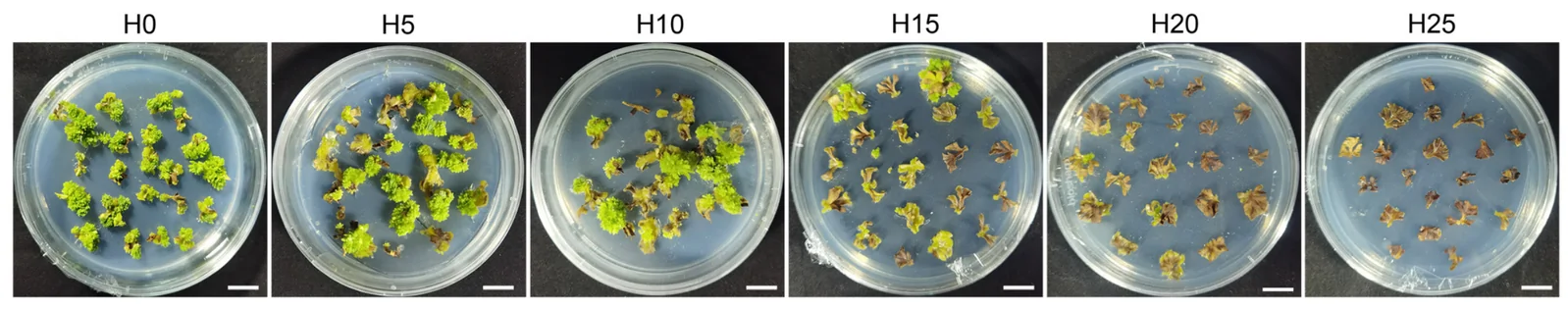
Effect of Hygromycin on leaf differentiation of *A. achilleoides* (30 d), bars = 1 cm.

Hygromycin is a commonly used selective antibiotic for screening transformed explants. In medium without hygromycin, explants were able to normally form calli and regenerate adventitious shoots. As hygromycin concentration increased, both callus induction and adventitious shoot differentiation rates gradually decreased, indicating that the regenerative capacity of explants was significantly inhibited (Table 7; Figure 5).

At 5 mg/L hygromycin, explants still maintained relatively high regenerative ability. When the concentration increased to 10–15 mg/L, regeneration efficiency was markedly reduced. At 20 mg/L, explant regeneration was severely restricted, and at 25 mg/L, both callus induction and adventitious shoot differentiation were almost completely inhibited (Table 7, Figure 5). Therefore, 25 mg/L hygromycin was determined as the critical selective pressure for leaf explants of *A. achilleoides*.

**Figure 6:**
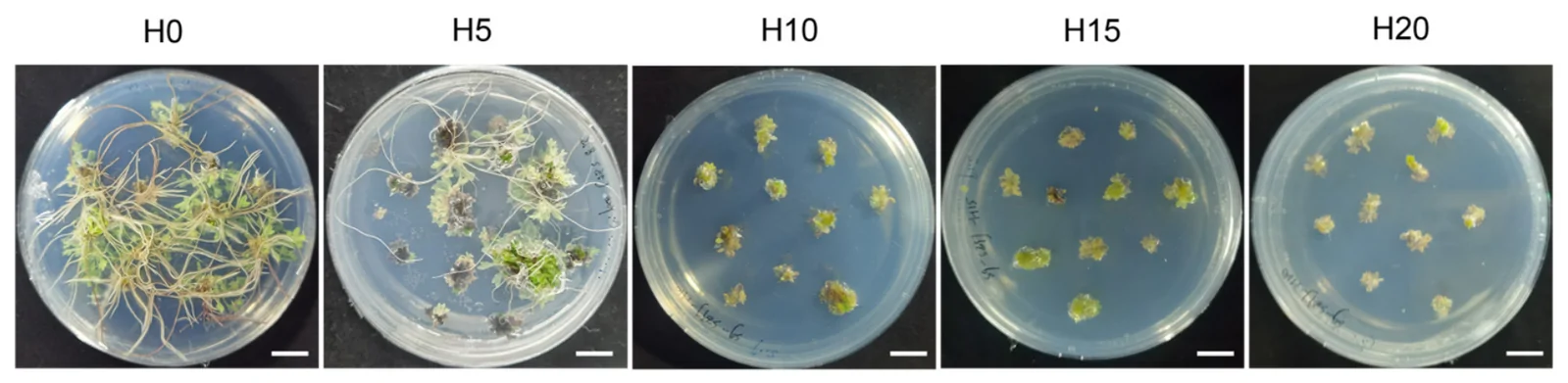
Effect of hygromycin on rooting of adventitious buds of *A. achilleoides* (30 d), adventitious shoots (1–2 cm in length) were transferred to rooting medium containing different concentrations of hygromycin. bars = 1 cm.

Excised adventitious shoots (1–2 cm in length) transferred to rooting medium without hygromycin developed normal roots. However, with increasing hygromycin concentration, the rooting rate decreased significantly. When the concentration reached 10 mg/L or higher, rooting was completely inhibited, and plantlets showed obvious browning. Accordingly, 10 mg/L hygromycin was selected as the critical selective pressure for rooting of adventitious shoots of *A. achilleoides* (Table 8, Figure 6).

#### 3.2.2. Establishment of an Agrobacterium-Mediated Genetic Transformation System for *A. achilleoides*

Pre-culture duration, *Agrobacterium* suspension density, infection duration, and co-cultivation period are critical factors affecting plant genetic transformation efficiency. To optimize the transformation procedure, these factors were evaluated using an orthogonal experimental design. After 30 days of culture, transformation performance was assessed based on resistant shoot induction rate, differentiation rate, adventitious shoot formation coefficient, and browning rate (Table 9).

##### Effect of Pre-Culture Duration on Resistant Shoot Regeneration

Pre-culture duration significantly affected the induction and differentiation of resistant shoots in *A. achilleoides* (Table 10). As pre-culture duration increased, the differentiation rate of resistant shoots initially decreased and subsequently increased. The differentiation rates obtained after 0 and 3 days of pre-culture were 35.01% and 36.08%, respectively, which were significantly higher than those observed after 1 and 2 days of pre-culture. In contrast, the adventitious shoot formation coefficient showed no significant differences among treatments, ranging from 1.53 to 1.64.

The browning rate varied significantly with pre-culture duration, reaching its highest value at 2 days (37.78%) and its lowest value at 3 days (17.31%). These results indicate that a 3-day pre-culture period provides a favorable physiological state for explants, resulting in enhanced shoot differentiation and reduced tissue browning.

##### Effect of Agrobacterium Suspension Density on Resistant Shoot Regeneration

Different *Agrobacterium* suspension densities significantly affected both resistant shoot differentiation and tissue browning (Table 11). As the bacterial density increased from OD_600_ = 0.2 to 0.6, the differentiation rate increased significantly. The highest differentiation rate (40.66%) was obtained at OD_600_ = 0.6, which was significantly higher than those at OD_600_ = 0.4 and 0.8. Moreover, the browning rate at this density was only 19.53%, significantly lower than that observed at OD_600_ = 0.2 and 0.4.

Further increasing the bacterial density to OD_600_ = 0.8 resulted in a significant decline in differentiation rate to 18.40%, accompanied by a reduction in the adventitious shoot formation coefficient to 1.27. Although OD_600_ = 0.2 produced the highest shoot formation coefficient (1.95), its differentiation rate was lower than that achieved at OD_600_= 0.6. These findings suggest that a moderate Agrobacterium density is conducive to efficient resistant shoot regeneration.

##### Effect of Infection Duration on Resistant Shoot Regeneration

Infection duration significantly influenced both the differentiation rate and adventitious shoot formation coefficient of resistant shoots (Table 12). At an infection duration of 5 min, the differentiation rate was 26.64%, and the shoot formation coefficient was 1.48. The lowest differentiation rate (17.93%) was observed at 10 min. Increasing the infection duration to 20 min improved the differentiation rate to 33.41%, with a shoot formation coefficient of 1.69. The highest differentiation rate (40.19%) was obtained after 30 min of infection, with a shoot formation coefficient of 1.50.

The browning rate increased progressively with longer infection durations, ranging from 19.01% at 10 min to 34.64% at 30 min. These results demonstrate that infection duration plays a critical role in resistant shoot regeneration. Although a 30 min infection period produced the highest differentiation rate, it also increased tissue browning, indicating that its overall effect should be evaluated in combination with other transformation factors.

##### Effect of Co-Cultivation Duration on Resistant Shoot Regeneration

Co-cultivation duration significantly affected resistant shoot induction, differentiation, and tissue browning (Table 13). Differentiation rates of 37.50% and 42.08% were obtained after 1 and 2 days of co-cultivation, respectively, both of which were significantly higher than those observed after 3 and 4 days.

The highest adventitious shoot formation coefficient (1.86) was recorded following 1 day of co-cultivation, whereas extending the co-cultivation period to 3–4 days markedly reduced shoot regeneration capacity. Tissue browning increased significantly with prolonged co-cultivation, reaching 43.19% and 36.95% after 3 and 4 days, respectively, compared with only 14.13% and 16.05% after 1 and 2 days.

Therefore, a co-cultivation period of 1–2 days appears optimal for maintaining high regeneration efficiency while minimizing explant browning.

##### Effects of Different Infection Conditions on Hygromycin-Resistant Shoot Formation

Range analysis revealed substantial differences in the relative contribution of the four factors to hygromycin-resistant shoot differentiation (Table 14). The factors were ranked as follows: co-cultivation duration (R = 22.27) > *Agrobacterium* suspension density (R = 22.26) ≈ infection duration (R = 22.26) > pre-culture duration (R = 10.89). These results indicate that co-cultivation duration, bacterial density, and infection duration were the principal factors affecting the formation of hygromycin-resistant shoots, whereas pre-culture duration exerted a comparatively smaller effect.

Single-factor analysis showed that a pre-culture duration of 3 days, bacterial density of OD_600_ = 0.6, infection duration of 30 min, and co-cultivation period of 2 days individually corresponded to relatively high differentiation rates. However, the orthogonal experiment (Table 9) demonstrated that Treatment 13, consisting of 3 days of pre-culture, OD_600_ = 0.2, 30 min infection, and 2 days of co-cultivation, achieved the highest resistant shoot differentiation rate (62.78%), which was significantly higher than those of the other 15 treatments. In addition, this treatment yielded a resistant shoot induction rate of 97.41% and a relatively low browning rate of 19.20%, indicating a favorable parameter combination for generating hygromycin-resistant shoots. The difference between the single-factor and orthogonal results likely reflects the interaction among transformation parameters. In the single-factor experiment, *Agrobacterium* density was evaluated independently, whereas in the orthogonal design, bacterial density was assessed together with pre-culture duration, infection duration, and co-cultivation period. Therefore, OD_600_ = 0.2 may have provided sufficient bacterial contact for T-DNA delivery while reducing bacterial stress under the combined conditions of Treatment 13.

Taken together, the results of both the single-factor and orthogonal experiments identified Treatment 13 as the most suitable parameter combination for generating hygromycin-resistant shoots of *A. achilleoides* under the conditions used in this study. Because hygromycin resistance alone cannot completely represent stable transformation events, resistant plantlets obtained from Treatment 13 were further subjected to molecular identification. PCR and sequencing analyses confirmed three independent double-positive transgenic lines carrying both *AtEC1.2* and *PpPAR* fragments, supporting the feasibility of Treatment 13 for obtaining molecularly confirmed transformants.

#### 3.2.3. Identification of Transgenic *A. achilleoides* Plants

**Figure 7:**
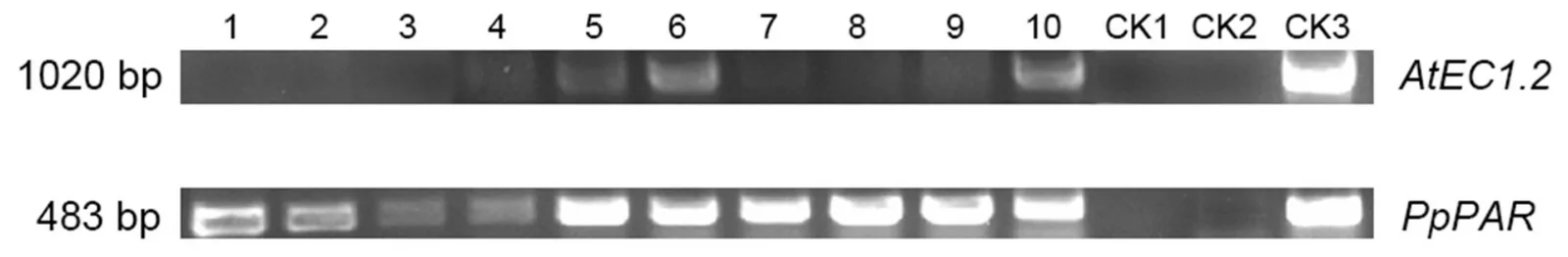
PCR amplification on the resistant plantlets of *A. achilleoides*. 1–10: Resistant plantlets; CK1: ddH2O; CK2: Wild type; CK3: Plasmid.

A total of 10 hygromycin-resistant plantlets were obtained from Treatment 13 of the orthogonal transformation experiment (3 days of pre-culture, OD_600_ = 0.2, 30 min infection, and 2 days of co-cultivation) following selection on medium containing 25 mg/L hygromycin and 350 mg/L timentin. Genomic DNA from these resistant lines was subjected to PCR analysis. All 10 resistant plantlets yielded the expected *PpPAR* amplification product, consistent with the positive control. Among these hygromycin-resistant plantlets, three independent lines showed amplification of both AtEC1.2 and PpPAR fragments, indicating double-positive transformation events. No target bands were detected in the wild-type control or the ddH2O negative control (Figure 7). The successful integration of both *AtEC1.2* and *PpPAR* fragments into the genome of *A. achilleoides* was confirmed in the three double-positive transgenic lines by PCR and sequencing analyses. The sequencing chromatograms showed clear and high-quality peaks, and sequence alignment analysis demonstrated that the amplified fragments were consistent with the expected *AtEC1.2* promoter and *PpPAR* gene sequences (Supplementary Figures S1 and S2). These results further confirmed the presence of the target transgenes in the three double-positive transgenic lines. Three independent double-positive transgenic lines were identified, representing a molecular confirmation rate of 30% among the hygromycin-resistant plantlets.

## 4. Discussion

### 4.1. Effects of Plant Growth Regulators on In Vitro Regeneration of A. achilleoides

The type and concentration of plant growth regulators are key determinants of in vitro regeneration efficiency [26,27,28,29]. In this study, NAA exerted a stronger effect than 6-BA on both callus induction and adventitious shoot differentiation in *A. achilleoides*, indicating that auxin levels are crucial for regulating the regenerative capacity of this species. As NAA concentration increased, callus induction rates significantly improved and remained high within the 0.8–1.0 mg/L range, suggesting that an optimal auxin concentration promotes cellular dedifferentiation and callus formation from leaf explants. Similar responses to appropriate NAA-containing media have been reported in cut-flower chrysanthemum (*Dendranthema × grandiflorum*), where BAP combined with NAA promoted callus induction [30], and in chamomile (*Matricaria chamomilla* L.), where NAA-kinetin combinations induced embryogenic callus formation [31]. In the closely related species *Aj. alabasica*, 1.0 mg/L 6-BA combined with 1.0 mg/L NAA was also suitable for callus induction and adventitious shoot differentiation [13]. These findings support the present result that an appropriate NAA level is important for promoting callus induction from leaf explants of *A. achilleoides*.

Adventitious shoot differentiation represents a critical stage in in vitro regeneration, marking the transition from cellular dedifferentiation to organogenesis, which is tightly regulated by the balance between cytokinins and auxins [32,33,34,35]. In this study, the highest adventitious shoot differentiation rate (100%) was achieved on medium containing 2 mg/L 6-BA and 1 mg/L NAA. Further increases in 6-BA concentration significantly reduced shoot regeneration efficiency. Similarly, Naing et al. [36] reported that excessively high 6-BA concentrations negatively affected shoot formation in chrysanthemum ‘Vivid Scarlet’. These results indicate that adventitious shoot regeneration in *A. achilleoides* also depends on a coordinated balance between cytokinins and auxins, and that excessive cytokinin may disrupt endogenous hormonal homeostasis, thereby reducing organogenic potential [37,38].

Rooting represents a crucial step in generating complete plants in vitro. In the present study, 1/2 MS medium was superior to full-strength MS for root induction, indicating that reduced salt concentration favors root development in *A. achilleoides*. Among the IBA-containing treatments, 1/2 MS medium supplemented with 0.5 mg/L IBA produced a 100% rooting rate, indicating that IBA is suitable for the rooting stage of regenerated shoots in A. achilleoides. IBA has been reported to effectively stimulate adventitious root formation and root system development [39,40]. Similar use of IBA for rooting has also been reported in other Asteraceae species, including *Chrysanthemum morifolium* [41], *Chrysanthemum* ‘Shinma’ [8], and *Chrysanthemum naktongense* [42], suggesting that IBA may also be suitable for rooting induction in other Asteraceae species.

### 4.2. Effects of Agrobacterium Infection Conditions on Genetic Resistant Shoot Regeneration and Transformation Performance

The establishment of an efficient genetic transformation system relies on a stable regeneration protocol and appropriate *Agrobacterium* infection conditions. In this study, pre-culture duration, bacterial suspension density, infection time, and co-cultivation period all significantly influenced transformation efficiency, highlighting the sensitivity of *A. achilleoides* explants to *Agrobacterium* infection parameters.

An appropriate pre-culture period may help explants adapt to the in vitro culture environment and improve their responsiveness to *Agrobacterium* infection and selection-based regeneration. Excessively long pre-culture may lead to tissue aging and browning, which is detrimental to subsequent transformation [43]. Single-factor analysis showed that 0 d and 3 d pre-culture produced high shoot differentiation rates, suggesting that moderate pre-culture fosters a cell state favorable for T-DNA integration, consistent with reports in chrysanthemum ‘Shinma’, where pre-culture duration significantly affects transformation efficiency [44].

Bacterial density and infection duration are key determinants of *Agrobacterium*-mediated transformation efficiency. Appropriate levels of both factors significantly enhance the recovery of resistant shoots, whereas overly weak or strong infection reduces transformation success [45,46]. Single-factor analysis indicated that a bacterial suspension at OD_600_ = 0.6 yielded the highest adventitious shoot differentiation rate, while a 30 min infection also produced maximal differentiation. In chrysanthemum ‘Jimba’, Fu et al. [47] observed the highest transformation efficiency at OD_600_ = 0.5 and 10 min infection. Differences in optimal infection conditions across studies may reflect variations in plant material, explant type, and bacterial strain [48]. In this study, although 30 min infection achieved high differentiation rates, browning was also notably increased, indicating that while prolonged contact between *Agrobacterium* and explants enhances T-DNA delivery, excessive bacterial adherence can trigger strong plant defense responses, reducing explant viability [49]. Therefore, infection conditions must balance transformation efficiency with tissue survival.

Co-cultivation is critical for T-DNA transfer and integration. Here, co-cultivation exceeding 2 d caused significant browning and necrosis, markedly reducing regeneration capacity. Previous studies have shown that prolonged co-cultivation promotes continued bacterial proliferation, exacerbates tissue stress and cellular damage, and consequently inhibits organogenesis and plant regeneration [50,51]. Thus, under the conditions of this study, 2 d co-cultivation was optimal, ensuring high transformation efficiency while minimizing the negative impact of excessive bacterial growth on explants.

Range analysis revealed that the impact of different factors on transformation efficiency varied, and the optimal single-factor level did not fully coincide with the optimal combination identified in the orthogonal experiment. For example, OD_600_ =0.6 achieved the highest mean resistant shoot differentiation rate in single-factor tests, whereas the best combination in the orthogonal design used OD_600_ = 0.2. This difference does not necessarily indicate inconsistency between the two analyses, but reflects that the single-factor test evaluates the average effect of each factor, whereas the orthogonal experiment assesses the overall performance of specific multi-factor combinations. This indicates that transformation efficiency is governed by the synergistic effects of multiple factors rather than any single parameter [52,53]. Under infection for 30 min and co-cultivation for 2 d, lower bacterial density was sufficient to achieve effective T-DNA delivery while reducing tissue damage and browning, resulting in a more favorable overall outcome. The most suitable combination was 3 d pre-culture, OD_600_ = 0.2, 30 min infection, and 2 d co-cultivation. This system maintained high resistant shoot differentiation while effectively controlling browning, providing a reliable technical foundation for *A. achilleoides* genetic transformation studies.

Although a stable *Agrobacterium*-mediated transformation system for *A. achilleoides* has been established, transformation efficiency can still be improved. Conventional infection relies mainly on bacterial entry through wound sites, and some target cells may have insufficient contact with *Agrobacterium*, potentially limiting T-DNA delivery. Studies have shown that vacuum infiltration and sonication-assisted *Agrobacterium*-mediated transformation (SAAT) can enhance bacterial contact with plant tissues, increasing transgene delivery efficiency [54,55]. Thus, combining these advanced infection techniques in future work could further optimize the genetic transformation system of *A. achilleoides,* providing a more efficient platform for functional analysis of drought- and other trait-related genes and molecular breeding applications.

### 4.3. Molecular Confirmation and Limitations of the Transformation System

Although Treatment 13 showed the best performance for generating hygromycin-resistant shoots based on resistant shoot regeneration, hygromycin resistance alone cannot confirm the presence of all introduced DNA fragments examined in this study. In the present study, only three of the 10 hygromycin-resistant plantlets were confirmed by PCR and sequencing to contain detectable fragments of both *AtEC1.2* and *PpPAR*, corresponding to a molecular confirmation rate of 30% among resistant regenerants. This result indicates that hygromycin-resistant plantlets require further molecular verification, because selection survival or amplification of a single fragment is insufficient to confirm that both the promoter-containing region and the target fragment have been retained.

The absence of *AtEC1.2* amplification in some *PpPAR*-positive lines may be associated with incomplete or rearranged T-DNA integration. During *Agrobacterium*-mediated transformation, T-DNA integration is a complex process, and the introduced T-DNA may not always be retained in the plant genome as an intact cassette. Previous studies have shown that T-DNA integration may involve deletions at T-DNA/plant DNA junctions, border-region truncation, partial insertion, or rearrangement of inserted fragments [56,57,58]. Therefore, detection of *PpPAR* indicates that part of the introduced cassette was present, but it does not necessarily confirm that the *AtEC1.2* promoter region was also retained. If the *AtEC1.2* promoter region was deleted, truncated, or rearranged, or if its primer-binding sites were disrupted during integration, *AtEC1.2* amplification could fail even when *PpPAR* remained detectable. Thus, simultaneous amplification and sequencing confirmation of both *AtEC1.2* and *PpPAR* provide stronger evidence for molecularly confirmed candidate lines than hygromycin resistance or single-fragment amplification alone.

It should also be noted that PCR and sequencing verify the presence and sequence identity of the introduced DNA fragments at the genomic level, but they do not demonstrate transcriptional activity or functional expression. RT-qPCR can be used to assess transcript accumulation [59], whereas reporter gene assays such as GFP and GUS are widely used to examine promoter activity and expression patterns in transgenic plants [60,61]. Therefore, the present study focused on establishing an *Agrobacterium*-mediated transformation procedure and verifying the presence of introduced fragments, whereas the transcriptional activity and functional performance of the introduced fragments remain to be further examined. Future studies should further verify whether the introduced fragments are transcriptionally active and functional using RT-qPCR and reporter gene assays such as GUS or GFP. Despite these limitations, the recovery of three double-positive lines indicates that the established protocol provides a preliminary and feasible basis for genetic transformation and further validation in this species.

## 5. Conclusions

A leaf-derived in vitro morphogenesis system and a preliminary *Agrobacterium* tumefaciens-mediated transformation procedure were successfully established for *A. achilleoides.* MS medium supplemented with 2.0 mg/L 6-BA and 1.0 mg/L NAA was optimal for adventitious shoot differentiation, while 1/2 MS medium containing 0.5 mg/L IBA was most suitable for rooting. For transformation optimization, the combination of a 3-day pre-culture period, infection with *A. tumefaciens* strain EHA105 at OD_600_ = 0.2 for 30 min, and 2 days of co-cultivation resulted in the highest hygromycin-resistant shoot regeneration performance. Following molecular analysis, three independent double-positive lines carrying both *AtEC1.2* and *PpPAR* fragments were confirmed by PCR and sequencing. Although further analyses are required to evaluate transgene expression and functional activity, the established system provides a foundation for future genetic transformation studies and molecular breeding applications in *A. achilleoides*.

## Figures and Tables

**Table 1:** The medium for rooting of *A. achilleoides*.

Treatment	Basal Medium	NAA (mg/L)	Indole-3-Butyric Acid (IBA) (mg/L)
G1	MS	/	/
G0	1/2 MS	/	/
G01		0.1	/
G02		0.3	/
G03		0.5	/
G04		/	0.1
G05		/	0.3
G06		/	0.5

**Table 2:** Selected factors and levels of the genetic transformation system in leaves of *A. achilleoides*.

Level	Factors			
	Pre-Culture Time (d)	Concentration of *Agrobacterium tumefaciens* (OD_600_)	Infection Time (min)	Co-Culture Time (d)
1	0	0.2	5	1
2	1	0.4	10	2
3	2	0.6	20	3
4	3	0.8	30	4

**Table 3:** Primer sequences used for PCR detection of AtEC1.2 and PpPAR in transgenic *A. achilleoides* plants.

Target Gene	Primer Name	Sequence (5′–3′)
*AtEC1.2*	*AtEC1.2*-F	AAATGTTCCTCGCTGACGTA
*AtEC1.2*	*AtEC1.2*-R	ACTTGTGTTAGAAGCCATTATTCTTTCT
*PpPAR*	*PpPAR*-F	ATGGTAGATGATGGCACCGC
*PpPAR*	*PpPAR*-R	TTCAGGCACCGTCGTCCTCCTCCG

**Table 4:** Effects of different medium settings on callus induction and adventitious bud differentiation from leaves of *A. achilleoides*.

Treatment	6-BA (mg/L)	NAA (mg/L)	Callus Induction Rate (%)	Differentiation Rate (%)
1	1.0	0.5	78.42 ± 20.88 bc	82.31 ± 15.71 bc
2	1.0	0.8	100.00 ± 0.00 a	91.11 ± 5.74 ab
3	1.0	1.0	100.00 ± 0.00 a	89.05 ± 8.61 ab
4	2.0	0.5	76.86 ± 18.98 c	55.00 ± 10.00 cd
5	2.0	0.8	100.00 ± 0.00 a	93.33 ± 7.64 ab
6	2.0	1.0	100.00 ± 0.00 a	100.00 ± 0.00 a
7	3.0	0.5	66.67 ± 36.17 c	22.04 ± 24.43 d
8	3.0	0.8	96.67 ± 5.77 ab	93.33 ± 7.64 ab
9	3.0	1.0	100.00 ± 0.00 a	95.00 ± 5.00 a

Note: Different lowercase letters in the same column indicate significant differences among different treatments (*p* < 0.05).

**Table 5:** Range analysis for callus induction and adventitious bud differentiation from leaves of *A. achilleoides* under different culture medium formulations.

	Callus Induction Rate (%) (6-BA)	Callus Induction Rate (%) (NAA)	Differentiation Rate (%) (6-BA)	Differentiation Rate (%) (NAA)
*K*_1_	835.25	682.02	797.35	564.95
*K*_2_	830.71	890.00	745.00	838.33
*K*_3_	795.00	888.95	771.11	810.18
*k*_1_	92.81	75.78	88.59	62.77
*k*_2_	92.30	98.89	82.78	93.15
*k*_3_	88.33	98.77	85.68	90.02
R	4.47	23.11	5.82	30.38

Note: *K_i_* represents the sum of experiments of this factor at level *i*, and *k_i_* represents the mean value of the factor in the case of level i, which is used to calculate the range R value. Level *i* is in the same Table 4.

**Table 6:** Effects of different medium settings on rooting of adventitious bud of *A. achilleoides*.

Treatment	Basal Medium	NAA (mg/L)	IBA (mg/L)	Root Length (cm)	Number of Roots	Rooting Rate (%)
G1	MS	/	/	7.57 ± 0.21 bc	6.00 ± 4.58 d	83.33
G0	1/2 MS	/	/	6.90 ± 2.43 c	8.94 ± 2.43 cd	100.00
G01		0.1	/	10.40 ± 0.10 a	9.44 ± 0.51 cd	94.44
G02		0.3	/	8.63 ± 0.51 b	11.44 ± 1.28 c	100
G03		0.5	/	5.50 ± 0.40 d	13.33 ± 3.06 a	100
G04		/	0.1	6.70 ± 0.20 c	19.67 ± 2.52 b	96.30
G05		/	0.3	7.50 ± 0.26 bc	19.33 ± 5.13 b	100.00
G06		/	0.5	9.07 ± 0.35 ab	18.33 ± 5.51 b	100.00

Note: Different lowercase letters in the same column indicate significant differences among different treatments (*p* < 0.05).

**Table 7:** Effects of different concentrations of Hygromycin on callus induction and adventitious bud differentiation from leaves of *A. achilleoides*.

Treatment	Hygromycin Concentrations (mg/L)	Callus Induction Rate (%)	Differentiation Rate (%)
1	5	96.67 ± 2.89 a	82.18 ± 2.50 b
2	10	92.42 ± 2.52 b	61.48 ± 1.31 c
3	15	70.41 ± 0.90 c	30.51 ± 2.61 d
4	20	30.20 ± 3.03 d	14.89 ± 2.05 e
5	25	0.86 ± 1.43 e	0.00 ± 0.00 f
CK	0	100.00 ± 0.00 a	100.00 ± 0.00 a

Note: Different lowercase letters in the same column indicate significant differences among different treatments (*p* < 0.05).

**Table 8:** Effects of different concentrations of Hygromycin on rooting of adventitious bud of *A. achilleoides*.

Treatment	Hygromycin Concentrations (mg/L)	Rooting Rate (%)
1	5	67.09 ± 4.32 b
2	10	0.00 ± 0.00 c
3	15	0.00 ± 0.00 c
4	20	0.00 ± 0.00 c
CK	0	100.00 ± 0.00 a

Note: Different lowercase letters in the same column indicate significant differences among different treatments (*p* < 0.05).

**Table 9:** Effect of different transformation conditions on the growth of resistant buds of *A. achilleoides*.

Treatment	Pre-Culture Time (d)	Concentration of *Agrobacterium tumefaciens* (OD_600_)	Infection Time (min)	Co-Culture Time (d)	Callus Induction Rate (%)	Differentiation Rate (%)	Coefficient of Adventitious Bud Production	Browning Rate (%)
1	0	0.2	5	1	93.68 ± 1.51 abc	48.71 ± 2.39 bc	2.43 ± 0.33 ab	4.96 ± 0.54 gh
2	0	0.4	10	2	89.08 ± 2.94 cd	29.96 ± 6.47 efg	1.76 ± 0.24 bc	14.10 ± 0.81 ef
3	0	0.6	20	3	83.73 ± 6.35 de	32.42 ± 5.60 defg	1.52 ± 0.02 c	41.57 ± 4.13 bc
4	0	0.8	30	4	85.24 ± 3.02 cde	28.97 ± 8.13 fg	0.67 ± 0.33 d	45.12 ± 2.44 b
5	1	0.2	10	3	54.05 ± 2.11 f	4.92 ± 0.08 i	2.00 ± 1.00 abc	40.33 ± 2.12 bcd
6	1	0.4	5	4	70.13 ± 4.14 e	14.93 ± 0.47 hi	1.07 ± 0.07 cd	45.60 ± 4.71 b
7	1	0.6	30	1	90.67 ± 4.81 bc	58.67 ± 3.53 ab	1.71 ± 0.15 bc	11.88 ± 1.58 fg
8	1	0.8	20	2	83.09 ± 0.24 de	43.20 ± 2.04 cde	1.78 ± 0.23 bc	13.24 ± 1.93 f
9	2	0.2	20	4	81.83 ± 5.86 de	26.22 ± 3.47 g	1.75 ± 0.07 bc	47.48 ± 1.62 b
10	2	0.4	30	3	67.87 ± 6.79 e	31.33 ± 6.84 defg	1.48 ± 0.02 c	62.37 ± 3.71 a
11	2	0.6	5	2	91.78 ± 2.99 bc	32.37 ± 2.71 defg	1.33 ± 0.17 c	17.67 ± 1.52 e
12	2	0.8	10	1	98.33 ± 1.67 ab	10.84 ± 1.21 hi	1.57 ± 0.47 c	26.67 ± 4.41 d
13	3	0.2	30	2	97.41 ± 2.59 ab	62.78 ± 6.96 a	1.63 ± 0.14 bc	19.20 ± 2.22 e
14	3	0.4	20	1	97.22 ± 2.78 ab	31.79 ± 6.33 defg	1.72 ± 0.36 bc	12.99 ± 2.21 fg
15	3	0.6	10	4	86.06 ± 4.80 cd	39.19 ± 4.25 def	1.93 ± 0.30 abc	4.59 ± 0.23 h
16	3	0.8	5	3	92.06 ± 1.15 abc	10.57 ± 1.58 hi	1.07 ± 0.07 cd	32.47 ± 2.18 c

Note: Different lowercase letters in the same column indicate significant differences among different treatments (*p* < 0.05).

**Table 10:** Effect of pre-culture time on the growth of resistant buds in *A. achilleoides*.

Pre-Culture Time (d)	Callus Induction Rate (%)	Differentiation Rate (%)	Coefficient of Adventitious Bud Production	Browning Rate (%)
0	87.93 ± 1.94 a	35.01 ± 3.12 ab	1.59 ± 0.18 ab	26.44 ± 4.21 b
1	74.49 ± 3.51 b	30.43 ± 4.54 b	1.64 ± 0.24 a	27.76 ± 3.88 b
2	84.95 ± 3.17 a	25.19 ± 2.65 b	1.53 ± 0.11 ab	37.78 ± 4.90 a
3	93.18 ± 1.54 a	36.08 ± 4.71 a	1.59 ± 0.13 b	17.31 ± 2.64 c

Note: Different lowercase letters in the same column indicate significant differences among different treatments (*p* < 0.05).

**Table 11:** Effect of concentration of *Agrobacterium tumefaciens* on the growth of resistant buds in *A. achilleoides*.

Concentration of *Agrobacterium tumefaciens* (OD_600_)	Callus Induction Rate (%)	Differentiation Rate (%)	Coefficient of Adventitious Bud Production	Browning Rate (%)
0.2	81.74 ± 3.99 b	35.66 ± 5.11 a	1.95 ± 0.23 a	27.99 ± 3.99 a
0.4	81.08 ± 3.47 b	26.75 ± 2.98 b	1.51 ± 0.12 b	33.82 ± 5.21 a
0.6	88.06 ± 2.12 a	40.66 ± 3.22 a	1.62 ± 0.11 ab	19.53 ± 3.10 b
0.8	89.68 ± 1.53 a	18.40 ± 3.41 c	1.27 ± 0.16 c	29.38 ± 3.12 a

Note: Different lowercase letters in the same column indicate significant differences among different treatments (*p* < 0.05).

**Table 12:** Effect of infection time on the growth of resistant buds in *A. achilleoides*.

Infection Time (min)	Callus Induction Rate (%)	Differentiation Rate (%)	Coefficient of Adventitious Bud Production	Browning Rate (%)
5	86.92 ± 2.11 a	26.64 ± 3.95 bc	1.48 ± 0.15 b	25.17 ± 3.51 b
10	84.37 ± 4.02 a	17.93 ± 4.11 c	1.69 ± 0.26 ab	19.01 ± 3.22 c
20	86.18 ± 2.21 a	33.41 ± 2.45 ab	1.69 ± 0.08 ab	31.42 ± 3.77 a
30	83.08 ± 3.11 a	40.19 ± 4.67 a	1.50 ± 0.14 b	34.64 ± 5.10 a

Note: Different lowercase letters in the same column indicate significant differences among different treatments (*p* < 0.05).

**Table 13:** Effect of co-culture time on the growth of resistant buds in *A. achilleoides*.

Co-Culture Time (d)	Callus Induction Rate (%)	Differentiation Rate (%)	Coefficient of Adventitious Bud Production	Browning Rate (%)
1	94.86 ± 1.25 a	37.50 ± 5.12 a	1.86 ± 0.19 a	14.13 ± 2.11 c
2	87.84 ± 1.76 b	42.08 ± 3.90 a	1.65 ± 0.10 ab	16.05 ± 1.15 c
3	74.43 ± 4.11 c	19.81 ± 3.14 c	1.52 ± 0.16 b	43.19 ± 4.02 a
4	83.32 ± 2.98 b	27.11 ± 3.21 b	1.35 ± 0.18 c	36.95 ± 4.99 b

Note: Different lowercase letters in the same column indicate significant differences among different treatments (*p* < 0.05).

**Table 14:** Range analysis of the resistant shoot differentiation rate of *A. achilleoides* under different transformation conditions.

Factors	Pre-Culture Time (d)	Concentration of *Agrobacterium tumefaciens* (OD_600_)	Infection Time (min)	Co-Culture Time (d)
*K*_1_	420.17	428.19	319.72	450.02
*K*_2_	365.13	321.03	215.11	504.91
*K*_3_	302.26	487.93	400.88	237.71
*K*_4_	432.99	220.84	482.28	325.35
*k*_1_	35.01	35.68	26.64	37.5
*k*_2_	30.43	26.75	17.93	42.08
*k*_3_	25.19	40.66	33.41	19.81
*k*_4_	36.08	18.4	40.19	27.11
R	10.89	22.26	22.26	22.27

Note: *K*_i_ represents the sum of experiments of this factor at level *i*, and *k_i_* represents the mean value of the factor in the case of level i, which is used to calculate the range R value. Level *i* is in the same Table 3.

## Data Availability

The original contributions presented in this study are included in the article/Supplementary Material. Further inquiries can be directed to the corresponding author.

## Supplementary Materials

The following supporting information can be downloaded at: https://www.mdpi.com/article/10.3390/biotech15030059/s1. Figure S1: Sanger sequencing validation of the *AtEC1.2* promoter fragment in transgenic *Ajania achilleoides* plants; Figure S2: Sanger sequencing validation of the *PpPAR* fragment in transgenic *Ajania achilleoides* plants.
